# Electron Paramagnetic Resonance Studies of Irradiated Grape Snails (*Helix pomatia*) and Investigation of Biophysical Parameters

**DOI:** 10.3390/molecules28041872

**Published:** 2023-02-16

**Authors:** Aygun Nasibova, Rovshan Khalilov, Mahammad Bayramov, İslam Mustafayev, Aziz Eftekhari, Mirheydar Abbasov, Taras Kavetskyy, Gvozden Rosić, Dragica Selakovic

**Affiliations:** 1 Institute of Radiation Problems, Ministry of Science and Education Republic of Azerbaijan, AZ1143 Baku, Azerbaijan; 2 Department of Biophysics and Biochemistry, Baku State University, AZ1148 Baku, Azerbaijan; 3 Department of Biochemistry, Faculty of Science, Ege University, Izmir 35040, Turkey; 4 Institute of Molecular Biology & Biotechnologies, Ministry of Science and Education Republic of Azerbaijan, 11 Izzat Nabiyev, AZ1073 Baku, Azerbaijan; 5 Institute of Catalysis and Inorganic Chemistry, Ministry of Science and Education Republic of Azerbaijan, AZ1143 Baku, Azerbaijan; 6 Department of Biology and Chemistry, Drohobych Ivan Franko State Pedagogical University, 82100 Drohobych, Ukraine; 7 Department of Materials Engineering, The John Paul II Catholic University of Lublin, 20-950 Lublin, Poland; 8 Department of Physiology, Faculty of Medical Sciences, University of Kragujevac, 34000 Kragujevac, Serbia

**Keywords:** grape snails, stress factors, gamma radiation, magnetic properties, magnetic nanoparticles, EPR signals, radionuclide composition

## Abstract

A study of grape snails *(Helix pomatia)* using the electron paramagnetic resonance (EPR) spectroscopy method, where shells were exposed to ionizing gamma radiation, indicated that the effect of radiation up to certain doses results in the emergence of magnetic properties in the organism. The identification of the EPR spectra of the body and shell parts of the control and irradiated grape snails separately showed that more iron oxide magnetic nanoparticles are generated in the body part of the grape snail compared to the shells. A linear increase in free radical signals (g = 2.0023) in the body and shell parts of grape snails, and a non-monotonic change in the broad EPR signal (g = 2.32) characterizing iron oxide magnetic nanoparticles was determined depending on the dose of ionizing gamma radiation. Additionally, the obtained results showed that grape snails can be used as bioindicators for examining the ecological state of the environment. At the same time, the radionuclide composition of the body and shell parts of the grape snails and their specific activities were determined by CANBERRA gamma spectroscopy. The FTIR spectra of mucin, a liquid secreted by snails, were recorded.

## 1. Introduction

To date, some research has been carried out on various living systems that are affected by various stress factors [1,2,3,4]. Several studies have investigated the effects of ionizing gamma radiation, ultraviolet rays, drought, humidity, temperature, and other stress factors on living systems [5,6,7]. Previously, we performed different research on the effect of various stress factors (temperature, gamma radiation, UV radiation, etc.) on plant systems [8,9,10,11,12,13]. The mechanisms of action of stress factors on various types of tree and shrub plants taken from nature, on seedlings of seeds grown in laboratory conditions, and on chloroplasts isolated from the leaves of higher plants have been studied. In the current study, paramagnetism phenomena occurring in living systems under the influence of stress factors were evaluated.

In our comparative studies, the plants were derived from both ecologically clean and polluted areas, and then, using the EPR method, it was revealed that environmental pollution causes the generation of iron oxide magnetic nanoparticles in plants. The identification of EPR spectra of plants of the same species showed that the intensities of broad EPR signals (g = 2.32, ∆H = 320 G) characterizing iron oxide magnetic nanoparticles in plants growing in ecologically polluted areas are higher than the intensities of the corresponding signals of plants growing in clean areas [14,15]. At the same time, the study of sprouts of some plant seeds (corn (*Zea mays* L.), wheat (*Triticum* L.), and peas (*Cicer arietinum* L.)) irradiated with different doses of ionizing gamma radiation in laboratory conditions showed that the effect of gamma radiation up to certain doses causes the formation of iron oxide magnetic nanoparticles in plants. We have also provided the mechanism of occurrence of this event [10,16,17].

To confirm the results, we continued our research with animal organisms and investigated the effects of radiation factors on laboratory rats *(Wistar albino)* and grape snails with shells *(Helix pomatia)* [18,19,20,21].

Some biophysical parameters of grape snails (*Helix pomatia*), which belong to the phylum of mollusks and are commonly found in Absheron (Azerbaijan), were investigated. During the study of the effect of radiation factors on grape snails, it was determined that these factors cause the emergence of magnetic properties in them.

There are several reasons why grape snails with shells are of interest as research objects. First of all, they are distinguished by their high vitality. Thus, snails are very resistant to biological, physical, chemical, and radioactive stress [22,23,24]. Their blood–vascular systems are open. Snails carry hemocyanin, a protein containing copper molecules, dissolved in blood plasma. Grape snails live in lightly shaded gardens, vineyards, and open areas. Their day is spent hiding in a shell. They feed at night. They mainly eat the green parts of plants [25,26,27].

In our previous works, we have shown that magnetic nanoparticles, especially magnetite (Fe_3_O_4_) and maghemite (γ-Fe_2_O_3_) play an important role in the function of biological systems. These nanoparticles lead to the appearance of magnetic properties in natural systems and the formation of broad EPR signals, which we first discovered in plant leaves [28,29,30,31,32].

For this reason, the study of the mechanisms of formation of nanoparticles of biogenic origin in all living systems is of great interest. With this in mind, in further experiments, we chose grape snails as the research object.

## 2. Results and Discussion

While conducting research with grape snails, the effect of ionizing gamma radiation on young and old grape snails was studied separately. Note that the age of snails is determined by the size of their shells. Young and old grape snails were placed in plastic containers. Young grape snails were irradiated with doses of 200 Gy, 400 Gy, and 600 Gy, and old grape snails were irradiated with ionizing gamma radiation at doses of 150 Gy, 250 Gy, 400 Gy, 600 Gy, and 800 Gy. Control snails and snails that underwent irradiation with different doses of gamma radiation young and old grape snails were stored in special containers in laboratory conditions at a temperature of 22–25 °C for 60 days. During this period, they were fed the same amount of vegetables (carrots and cucumbers) and water. The grape snails were kept in a regime of 16 h of light and 8 h of darkness.

After 60 days, their shell and body parts were separated, dried at room temperature under natural conditions, and prepared for EPR studies.

**Figure 1:**
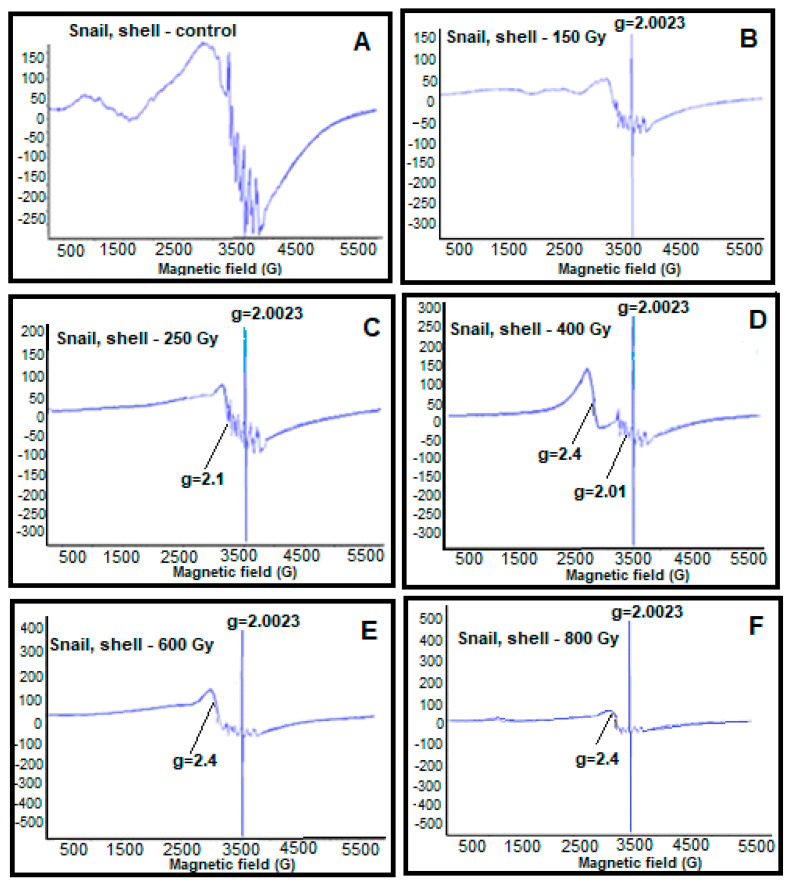
EPR spectra of shell parts of the control and irradiated at different doses of old snails. (**A**) control, (**B**) 150 Gy, (**C**) 250 Gy, (**D**) 400 Gy, (**E**) 600 Gy, and (**F**) 800 Gy.

Then, EPR spectra of control and irradiated samples were recorded in a wide range of magnetic fields (500–5500 G). It should be noted that consecutive measurements were performed at least five times. Figure 1 shows the EPR spectra of shell parts of the control and irradiated old snails at different doses.

The parameters of the EPR spectrometer are as follows: center field—3000 G; sweep width—5500 G; resolution—1024 points; frequency—9870 GHz; power—2102 mW; modulation frequency—100 kHz; modulation amplitude—10 G.

The identification of the EPR spectra of the shell parts of old grape snails recorded in Figure 1 illustrates the formation of free radical signals (g = 2.0023), broad EPR signals characterizing iron oxide magnetic nanoparticles (g = 2.4), six-component manganese ion signal with hyperfine structure (g = 2.01), and copper ions signals (g = 2.1). It was also observed that the increase in gamma radiation dose leads to a linear increase in the intensity of free radical signals (g = 2.0023). Depending on the dose of radiation, the monotonically dependent change in the intensity of the free radical signals obtained from the shell parts of grape snails allows us to use them as bioindicator parameters. Therefore, the regular change in the parameters of free radical signals depending on the dose of radiation allows us to use them in the assessment and monitoring of the ecological state of the environment.

Broad EPR signals characteristic of iron oxide magnetic nanoparticles (g = 2.4) appeared in the EPR spectra of the shell parts of grape snails. When the radiation was at 150 Gy, 250 Gy, we observed this signal at a small amplitude. Additionally, when the radiation dose reaches 400 Gy, the intensity of the broad EPR signal characterizing magnetic nanoparticles takes its maximum value. It has been found that irradiation of 600 Gy slightly reduces the intensity of this signal. Irradiation with ionizing gamma radiation at a dose of 800 Gy causes a doubling of the signal intensity.

Thus, as a result of the effect of radiation as a stress factor on snails in certain doses (150 Gy, 250 Gy, and 400 Gy), an increase in free iron ions in their body and the creation of a reducing environment occur. This leads to the formation of nanophase iron oxide particles as a result of biomineralization. However, although the escalation of radiation dose (600 Gy) increases free iron ions in the organism of the grape snails, the weakening of the reducing system decreases the formation of magnetic nanoparticles. The effect of radiation at the highest dose (800 Gy) results in the complete failure of the reducing system, and therefore the formation of nanoparticles does not occur. Thus, the absence of a reducing system leads to the generation of free radicals and reactive oxygen species (ROS). It is known that during stress, as a result of breaks in the living system, the bound iron changes to the free iron form. Due to the Fenton reaction, the increase in iron ions leads to the formation of ROS (for example, hydroxyl radical (HO^•^); superoxide anion (O2^•−^); hydrogen peroxide (H_2_O_2_); hydroxide ion (HO^−^), etc.). The living system transforms ROS into nano-sized magnetic particles to prevent them from multiplying. Additionally, the shells of snails do not have a reducing system. However, iron-based nanoparticles occur in them. This can be because the shells of grape snails are directly connected with the body and are “fed” through it. Thus, the body parts of grape snails affect the formation of the structure and composition of the shells’ parts.

**Figure 2:**
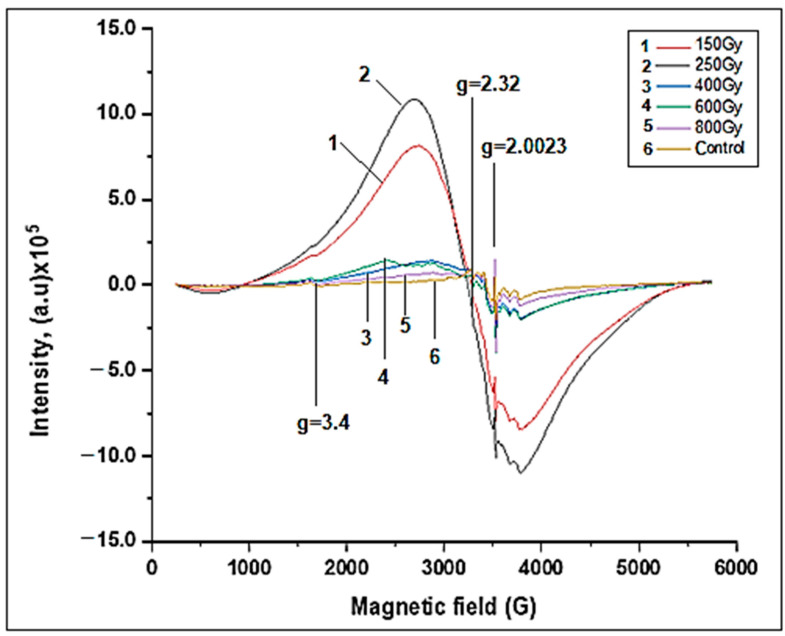
EPR spectra of body parts of old grape snails in the control and irradiated objects.

Figure 2 shows the EPR spectra of body parts of the control old grape snails and the old grape snails irradiated at different doses of gamma radiation. As shown, the spectra obtained from the body parts of snails show signals of free radicals (g = 2.0023), broad EPR signals characterizing iron oxide nanoparticles (g = 2.32), and signals of iron ions (g = 3.43). A comparison of the EPR spectra obtained from the shell and body parts of the grape snails shows that the intensity of the generated signals was more intense in the body samples. This may be due to the presence of a reducing system in the body parts of snails. Compared to the control, irradiation up to 250 Gy produces a broad EPR signal with a very high amplitude, which characterizes iron oxide magnetic nanoparticles. Increasing the radiation dose from 400 Gy to 800 Gy leads to a gradual fall in the intensity of this signal.

Certain doses (200 Gy and 350 Gy) of ionizing gamma radiation increase free iron ions in their body, and the presence of a reducing environment leads to the formation of nanophase iron oxide particles inside them as a result of biomineralization. Within the dose increment (600 Gy), again, the weakening of the reducing systems causes the formation of iron oxide magnetic nanoparticles to plummet there. Irradiation at the highest dose of gamma radiation (800 Gy) causes the complete breakdown of the reducing system, so the formation of nanoparticles does not occur. Thus, the absence of a reducing system leads to the formation of free radicals and ROS. This leads to the destruction of the living system.

This means that the formation of iron oxide magnetic nanoparticles as a result of stress has the purpose of self-defense of the organism.

**Figure 3:**
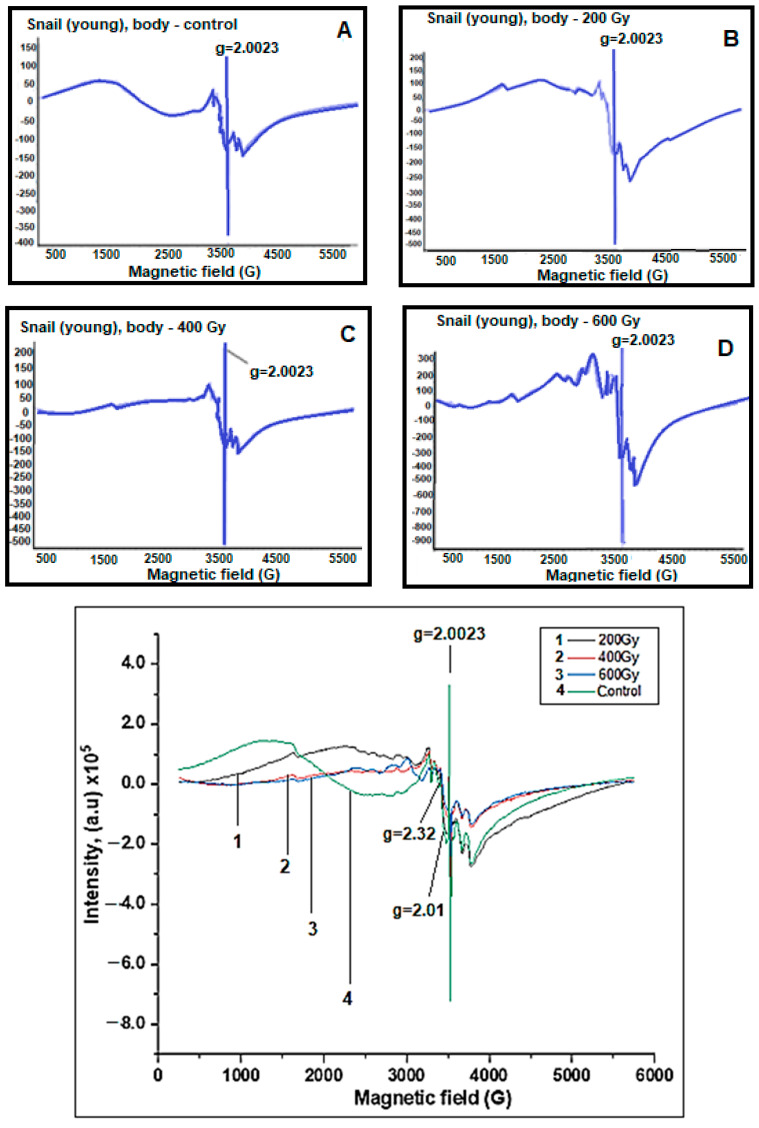
EPR spectra of body parts of the control and irradiated at different doses of young grape snails. (**A**) Control, (**B**) 200 Gy, (**C**) 400 Gy, (**D**) 600 Gy.

**Figure 4:**
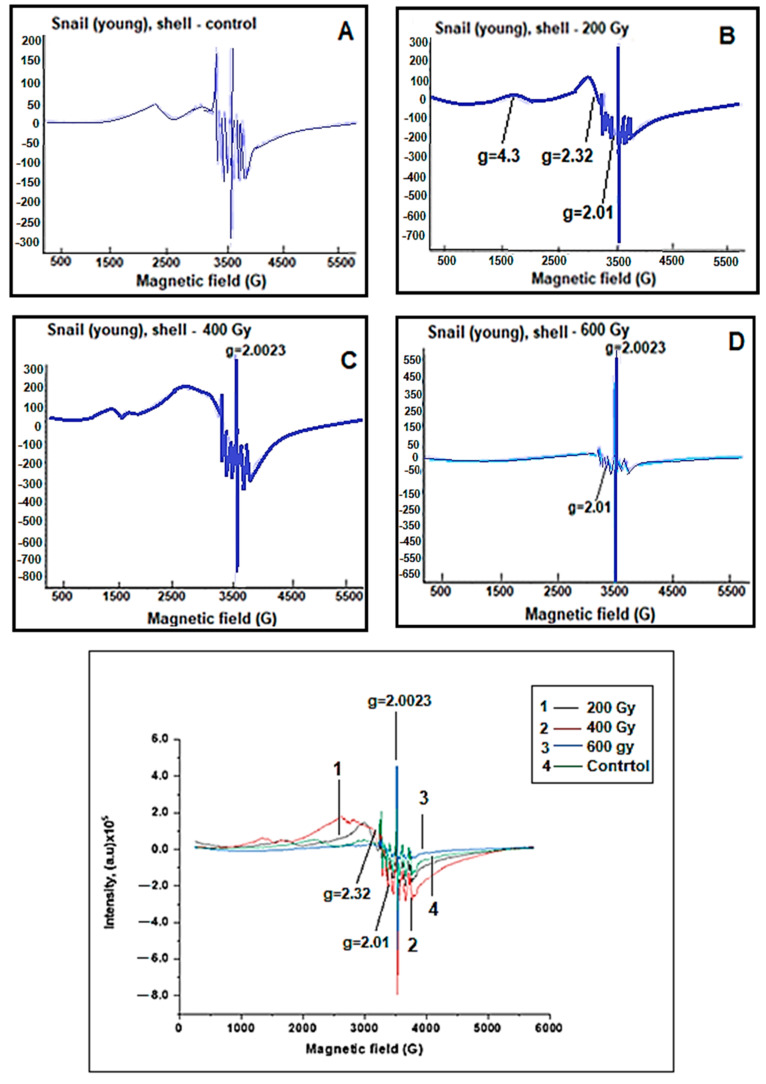
EPR spectra of shell parts of the control and irradiated with different doses of young grape snails. (**A**) Control, (**B**) 200 Gy, (**C**) 400 Gy, (**D**) 600 Gy.

EPR spectra of the body and shell parts of young grape snails were also recorded (Figure 3 and Figure 4). It was identified that the changes in the behavior of the EPR signals shell and body parts of old snails were also observed in the EPR spectra of young grape snails.

However, depending on the dose of gamma radiation in old grape snails, the dynamics of the changes in free radical signals (g = 2.0023) and characteristic broad EPR signals of iron oxide magnetic nanoparticles (g = 2.32) obtained from body parts were more intense than the dynamics of corresponding signals. This can be explained by the fact that old grape snails have lived longer in nature and have been more exposed to various environmental factors (for example, temperature, humidity, drought, UV radiation, and others).

**Figure 5:**
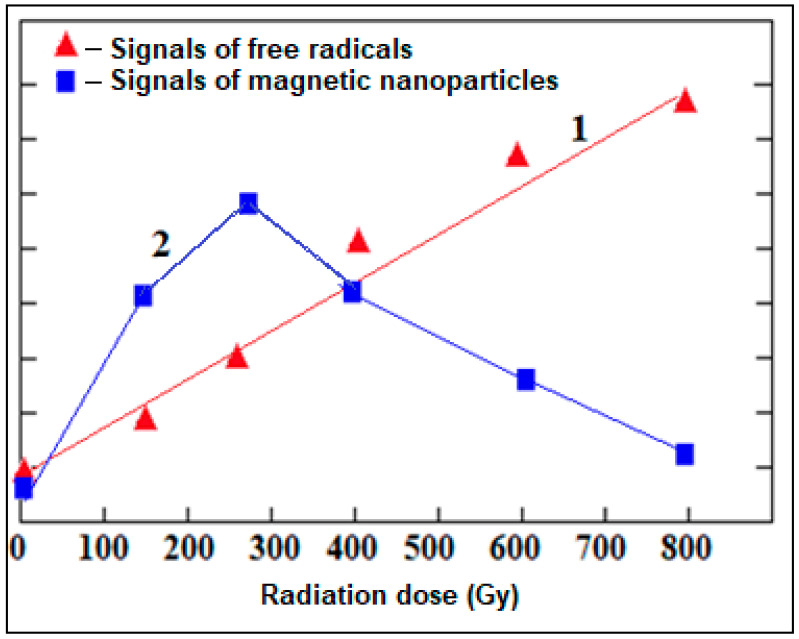
Dependence of the intensities of free radical signals (1) and broad EPR signals characterizing magnetic iron oxide nanoparticles (2) recorded in the body parts of grape snails on the radiation dose.

**Figure 6:**
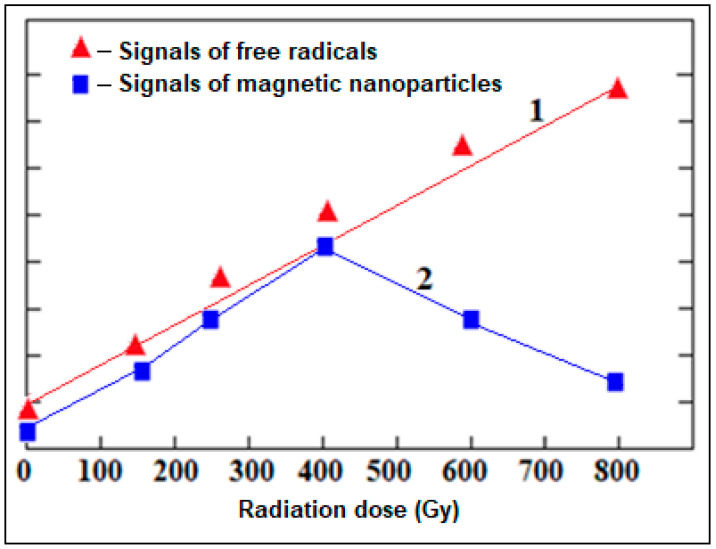
Dependence of the intensities of free radical signals (1) and broad EPR signals characterizing magnetic iron oxide nanoparticles (2) recorded in the shell parts of grape snails on the radiation dose.

The recorded spectra of the alterations of the intensity of the free radical signals and the intensity of the EPR signals of iron oxide magnetic nanoparticles depending on the radiation dose are shown graphically (Figure 5 and Figure 6). The obtained result is very important in monitoring and evaluating the ecological condition of the environment. We can say that the regular change in parameters of wide EPR signals characterizing iron oxide magnetic nanoparticles (g = 2.4) obtained from the body and shell parts of grape snails under the influence of stress factors is informative in assessing the degree of environmental pollution.

When studying the effect of various stress factors on living systems, it was found that stress factors cause the emergence of new magnetic properties in animal organisms as well as in plant systems.

Thus, during the study of the effect of various stress factors on living systems (plants, animal organisms, and chloroplasts isolated from higher plant leaves) using the EPR method, it was found that any stress factor in certain doses as a result of biomineralization causes the formation of iron oxide magnetic nanoparticles (magnetite—Fe_3_O_4_ and maghemite—γ—Fe_2_O_3_) in a living system. These nanoparticles lead to the formation of new magnetic properties in living systems. This result is very promising and relevant in terms of assessment and biomonitoring of the ecological state of the environment. Because our research showed that the parameters of EPR spectra of control and irradiated samples can be used as bioindicator parameters, these signals are informative in environmental monitoring.

In addition, the obtained results are also considered very important in terms of biomedical applications. Thus, our experiments showed that as a result of stress factors up to certain doses, nano-sized iron oxide particles are formed in living systems. These nanoparticles play an important role in medical treatment and diagnostics.

As a continuation of the experiments, the radionuclide composition of the bodies and shells of the studied grape snails and their specific activities were also determined (Table 1).

It was found that more radionuclides (^40^K, ^232^Th, 226Ra, ^228^Ra, ^137^Cs, ^235^U, ^238^U) are collected in the shell than in the body parts of grape snails. At the same time, higher specific activities of radionuclides were found in the shell. This can be explained by the fact that the shell is exposed to more stress factors than the body.

**Figure 7:**
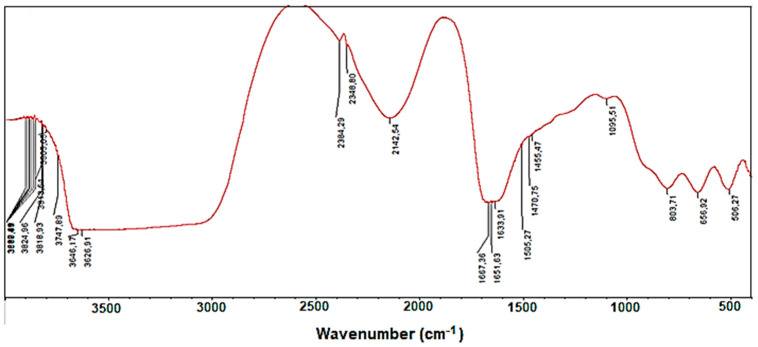
FTIR spectra of mucin secreted by grape snails.

Our research continued with the study of the mucin secreted by grape snails. Infrared spectra of mucin were recorded (see Figure 7).

In the spectra, the 3800–3300 wavenumbers belong to OH groups, 2142 to the absorption bands of C=C double bonds, 1667–1651 to the CO group, 803 (R)2 to the absorption band of out-of-plane deformation oscillations of C-H bonds connected to the double bond in the corner position of the C=CHR double bond, and 656, 508 to skeletal oscillations of C-C bonds.

## 3. Materials and Methods

In the conducted studies, young and old grape snails with shells collected from different areas of Absheron (Azerbaijan) were used as research objects. It should be noted that the age of snails is determined by the size of their shell parts. The shell color of grape snails is usually yellowish brown. Usually, they have wide stripes of dark brown color on their shells, but there are snails without them at all.

Snails are usually collected from nature in spring, summer, and early autumn. This is due to the hibernation of snails in cold seasons (at temperatures below 7 °C). Although the grape snail has a large shell and is slow, it can be a good digger. As soon as autumn comes, the snail digs a hole in the ground with its foot and then hibernates. If the ground cannot be dug due to the high density of the earth, the snail rolls over onto its back, scoops up more fallen leaves, and hibernates in this way. How long grape snails live is greatly influenced by their living conditions. In nature, this period is up to 8 years.

**Figure 8:**
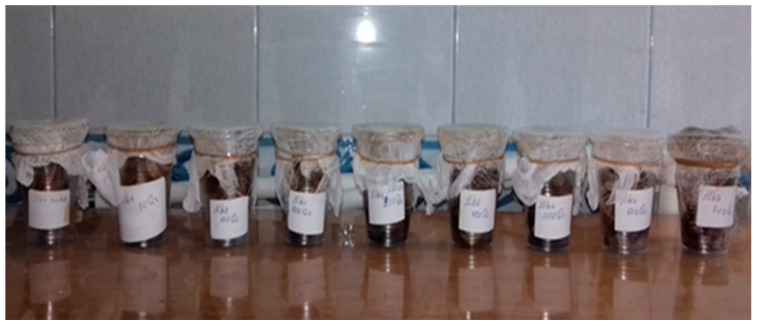
Control and irradiation with different doses of packaged grape snails.

The grape snails that are the objects of research were collected from nature in spring and summer. After the snails were placed in special containers with 20 individuals in each, they were irradiated with different doses of ionizing gamma radiation (young snails: 200 Gy, 400 Gy, and 600 Gy; old snails: 50 Gy, 250 Gy, 450 Gy, 600 Gy, and 800 Gy) in a “RUHUND—20000” device with a CO 60 source (Figure 8). For 60 days after irradiation, the life activities and feeding of snails were monitored, and their death rates were determined. It should be noted that during this period, the food ration of the snails was completely the same. It was revealed that the life activities and nutrition of snails weaken with a high dose of gamma radiation. At the same time, the increase in the radiation dose led to an increase in their mortality rate. Thus, it was determined that two individuals irradiated with a dose of 200 Gy died, three individuals irradiated with a dose of 400 Gy died, and five individuals irradiated with a dose of 800 Gy died. In general, it was found that snails are resistant to the effects of radiation factors.

**Figure 9:**
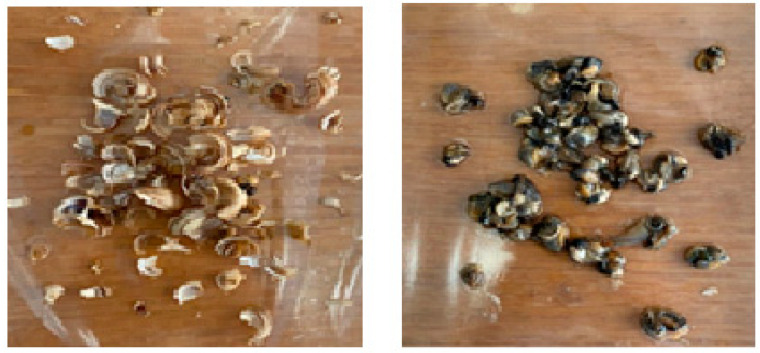
Shell and body parts of grape snails dried at room temperature.

After 60 days, the shells and body parts of the snails were separated and dried under natural conditions at room temperature (22–25 °C) (Figure 9) for 10–14 days. Spectra of the dried samples were recorded at room temperature on an Electron Paramagnetic Resonance Spectroscopy (EMX-BRUKER (Rheinstetten, Germany)).

In addition, the mucin secreted by grape snails with shells was studied by the infrared (IR) spectroscopy method (Figure 7). The quality of the extracted mucin depends on many factors: temperature, season, and diet of the snail. The study of snail mucin is of great interest. The snails release mucin during times of stress or injury. As a complicated biological complex, mucin increases the regeneration properties of the epidermis, enriches the cells of the deep layers of the skin with water, fights inflammation, and acts as a highly effective natural antioxidant. Mucin is also used for cosmetic products.

The radionuclide composition of grape snails’ bodies and shell parts and their specific activities were determined using CANBERRA gamma spectroscopy. This gamma spectroscopy is designed to measure the energies of X-ray or gamma radiation quanta emitted by radionuclides, as well as the activity (specific, volume) of gamma-emitting radionuclides in samples and objects. The results we obtained are shown in Table 1. For this purpose, when preparing the samples, the shell and body parts of the grape snails were separated from each other, dried at room temperature, and crushed by grinding. Then, the dried samples were placed in special Marinelli containers, and after some time (7–10 days), their radiospectrometric analysis was performed.

## 4. Conclusions

Using the EPR method, we studied new paramagnetic centers formed in living systems during the impact of various stress factors. The study of paramagnetic centers in various types of plant organisms has shown that the effect of stress factors causes the emergence of magnetic properties in them. In recent years, we have been conducting research on animal organisms to show the generality of the observed phenomenon. The presented work is dedicated to the study of new paramagnetic centers formed in old and young grape snails during the impact of ionizing gamma radiation, which is one of the stress factors.

The effect of ionizing gamma radiation on old and young grape snails was studied using the EPR method. The formation of paramagnetic centers was investigated in control grape snails and grape snails exposed to gamma radiation. It was found that gamma radiation causes the emergence of new magnetic properties in animal organisms, as well as plant systems.

EPR spectra of the body and shell parts of the control and exposure to various doses of gamma-irradiated young and old grape snails were recorded in a wide range of the magnetic field (500–5500 G). It was found that the intensity of the free radical signals (g = 2.0023) recorded in both the shell and body parts of grape snails increased linearly by elevating the gamma radiation dose. However, non-monotonic behavior of the intensities of the EPR signals (g = 2.32) characterizing the nanophase iron oxide particles was observed with the increase in the dose of gamma radiation in both the shell and the body parts. Therefore, the intensity of these signals was observed to increase gradually up to radiation doses of approximately 250–400 Gy, and gradually decrease during the subsequent increase of the radiation dose. In addition, it was determined that the amplitudes of the broad EPR signals characterizing iron oxide magnetic nanoparticles are more intense in the body parts of snails than in the shells. At the same time, a comparative study of old and young snails using the EPR spectroscopy method showed that the magnetic properties of old snails were higher than those of young snails. This can be explained by the fact that older snails are more exposed to various stress factors (temperature, UV radiation, drought, humidity, etc.).

The results obtained during the studies conducted with grape snails can be used in the assessment of the ecological status of the environment and many modern biomedical studies. The changes in the behavior of the EPR spectra of grape snails depending on the radiation dose show that these signals are informative in monitoring and evaluating the ecological state of the environment. Thus, depending on the dose of radiation, the regular change in the intensities of the free radical signals (g = 2.0023), as well as of the wide range of EPR signals characterizing iron oxide magnetic nanoparticles (g = 2.4) from the body and shell parts of snails, allows us to use snails as bioindicators.

The obtained results are also of practical importance in terms of applications in biomedicine, because in modern times, magnetic nanoparticles are widely used in medicine for both diagnostic and therapeutic purposes [33,34,35]. Additionally, we show in our research that magnetic properties are created in living systems during stress.

Thus, the study of paramagnetic centers in grape snails using the method of electron paramagnetic resonance spectroscopy showed that as a result of the impact of ionizing gamma radiation, which is one of the stress factors, nanophase magnetic iron oxide particles are formed in living systems.

It was determined that magnetite crystals in biological tissues are generated due to the phenomenon of biomineralization. The detection of iron oxide nanoparticles using EPR signals can be used as a new source of biochemical and biophysical information in biomedical research.

Determining the radionuclide composition of the shell and body parts of grape snails separately, it was concluded that the radionuclides in the shell have a higher specific activity. This is explained by the fact that the shell of snails is more exposed to stress than the body.

## Figures and Tables

**Table 1:** Radionuclide compositions of shell and body parts of grape snails and their specific activities.

Radionuclides	Unit of Measure	Grape Snail Shell	Grape Snail Body
^40^K	Bq/kg	26.1 ± 3.1	6.7 ± 1.5
^232^Th	Bq/kg	6.2 ± 0.3	MDA = 0.32
^226^Ra	Bq/kg	3.1 ± 0.5	MDA = 0.63
^228^Ra	Bq/kg	4.3 ± 0.4	MDA = 0.55
^137^Cs	Bq/kg	1.39 ± 0.12	0.91 ± 0.14
^235^U,	Bq/kg	0.08 ± 0.03	MDA = 0.03
^238^U	Bq/kg	1.65 ± 0.31	MDA = 0.65

## Data Availability

The data used to support the findings of this study are included in the article.

## Sample Availability

Samples of the compounds are available from the authors.
